# Autoantibody profiles and fibrosis indices across HBV-related disease stages: a retrospective laboratory-based study in a tertiary hospital in China

**DOI:** 10.1186/s12876-026-04718-4

**Published:** 2026-03-11

**Authors:** Hui Chen, Zhou Zhang, Shuo Jiang, Guojun Zheng, Zhen Zhu

**Affiliations:** 1 Center of Medical Laboratory, Changzhou Third People’s Hospital, Changzhou, Jiangsu China; 2 Department of Clinical Laboratory, Xishan People’s Hospital of Wuxi City, Xishan Clinical Medical School, Kangda College of Nanjing Medical University, Wuxi, Jiangsu China; 3 Central Laboratory, Xishan People’s Hospital of Wuxi City, Xishan Clinical Medical School, Kangda College of Nanjing Medical University, Wuxi, Jiangsu China

**Keywords:** Chronic hepatitis B, Antinuclear antibody, Autoantibodies, APRI, FIB-4, Liver fibrosis, Hepatocellular carcinoma.

## Abstract

**Background:**

Chronic hepatitis B (HBV) is frequently accompanied by immune dysregulation, and autoantibodies are intermittently detected in clinical practice. However, the clinical significance of autoantibody positivity across different HBV-related disease stages and its relationship with non-invasive fibrosis indices remains unclear.

**Methods:**

We retrospectively analyzed 151 patients with HBV-related liver disease, including chronic hepatitis B (CHB), liver cirrhosis (LC), and hepatocellular carcinoma (HCC). Antinuclear antibodies (ANA) and extended autoantibody profiles were evaluated together with the aspartate aminotransferase–to–platelet ratio index (APRI) and fibrosis-4 index (FIB-4). Clinical and laboratory parameters were compared across disease groups, and receiver operating characteristic (ROC) curves were constructed to assess the diagnostic performance of APRI and FIB-4 for identifying advanced liver disease.

**Results:**

ANA positivity increased progressively from CHB to LC and HCC, whereas extended autoimmune liver–specific autoantibodies remained rare across all stages. APRI and FIB-4 showed stepwise elevation with advancing disease severity, with FIB-4 demonstrating better diagnostic performance for advanced liver disease. ANA positivity showed no significant association with fibrosis indices, suggesting that these features may reflect distinct aspects of disease-related immune dysregulation.

**Conclusion:**

ANA positivity in chronic HBV infection appears to primarily reflect nonspecific immune activation rather than true autoimmune liver disease. FIB-4 provides practical and readily available information for identifying advanced liver disease, particularly in settings where liver biopsy or elastography is unavailable. Consideration of autoantibody patterns in conjunction with fibrosis indices may aid laboratory interpretation and support more informed clinical assessment in HBV-related liver disease.

**Supplementary Information:**

The online version contains supplementary material available at 10.1186/s12876-026-04718-4.

## Introduction

Chronic hepatitis B (CHB) affects nearly 300 million individuals worldwide and remains a major global health burden, with a well-recognized potential to progress from chronic hepatitis to liver cirrhosis (LC) and hepatocellular carcinoma (HCC) [1, 2]. The natural history of hepatitis B virus (HBV) infection is shaped by complex interactions among viral replication, host immune responses, and persistent hepatic inflammation.

Chronic HBV infection is frequently accompanied by immune dysregulation and has been reported to coexist with autoantibody positivity, even in the absence of overt autoimmune liver disease [3, 4]. Among these, antinuclear antibodies (ANA) are intermittently detected in patients with chronic viral hepatitis; however, their diagnostic and clinical relevance in HBV infection remains poorly defined. In particular, the significance of ANA positivity is controversial, as it may reflect nonspecific immune activation rather than true autoimmune liver disease [5, 6].

In contrast, extended autoimmune liver-specific autoantibodies, including antimitochondrial antibody M2 (AMA-M2), glycoprotein 210 (GP210), SP100, liver kidney microsomal antibody type 1 (LKM-1), and soluble liver antigen (SLA), are considered highly specific markers of autoimmune liver diseases. Available evidence suggests that these autoantibodies occur infrequently in patients with chronic HBV infection, underscoring the importance of cautious interpretation when such antibodies are detected in this population.

Parallel to immunological assessments, noninvasive fibrosis indices—most notably the aspartate aminotransferase–to–platelet ratio index (APRI) and the fibrosis index based on four factors (FIB-4)—have become essential laboratory tools for evaluating liver fibrosis and stratifying disease severity in chronic viral hepatitis [7, 8]. These indices serve as practical alternatives to liver biopsy in routine clinical practice, with FIB-4 demonstrating superior performance for identifying advanced fibrosis and predicting hepatocellular carcinoma risk in multiple HBV cohorts.

Despite these advances, few studies have systematically integrated autoantibody profiles with noninvasive fibrosis markers across clearly defined stages of HBV-related liver disease. In clinical practice, ANA positivity in patients with chronic hepatitis B often raises concern for concomitant autoimmune liver disease, while fibrosis stage represents an integrated indicator of disease severity and clinical risk. However, whether autoantibody positivity reflects autoimmune pathology or nonspecific immune activation accompanying disease progression remains unclear.

Therefore, by jointly evaluating autoantibody profiles and noninvasive fibrosis indices (APRI and FIB-4) across chronic hepatitis B, liver cirrhosis, and hepatocellular carcinoma, the present study aims to provide a practical framework for laboratory interpretation and support more informed clinical assessment in HBV-related liver disease.

## Materials and methods

### Study design and setting

This retrospective observational study was conducted at a Grade IIIA tertiary infectious disease hospital in China. Consecutive patients with confirmed chronic HBV infection admitted between January 2025 and October 2025 were screened for eligibility and enrolled during a predefined study period. No pre-selection of cases based on clinical characteristics, autoantibody status, or fibrosis indices was performed. The study protocol was approved by the institutional ethics committee, and the requirement for informed consent was waived due to the retrospective study design.

### Patient enrollment

#### Inclusion criteria

Patients were eligible if they met all of the following criteria:

1. Age ≥ 18 years.
2. Chronic HBV infection, defined as HBsAg positivity for ≥ 6 months.
3. Availability of complete laboratory data, including:
  ◦ ANA and extended autoantibody testing
  ◦ Liver biochemistry
  ◦ Complete blood count
  ◦ HBV-DNA quantification
4. A definitive clinical diagnosis of chronic hepatitis B (CHB), liver cirrhosis (LC), or hepatocellular carcinoma (HCC).

#### Exclusion criteria

To minimize immunologic and etiologic confounding, patients were excluded if they had:

- Co-infections such as HCV, HDV, HIV, or syphilis;
- Known autoimmune liver disease (e.g., autoimmune hepatitis or primary biliary cholangitis);
- Systemic autoimmune disorders (e.g., systemic lupus erythematosus, rheumatoid arthritis, Sjögren syndrome);
- Prior or current use of immunosuppressive therapy within 6 months;
- Pregnancy;
- Substantial missing data or incomplete autoantibody testing;
- Mixed-etiology liver disease (e.g., significant alcoholic liver disease, NASH requiring treatment, drug-induced liver injury).

Information on antiviral therapy was available for a subset of patients; however, due to heterogeneity in treatment regimens and duration, antiviral therapy status was not incorporated into group comparisons or statistical analyses.

### Diagnostic criteria

#### Chronic hepatitis B (CHB)

CHB was defined as HBsAg positivity for ≥ 6 months without clinical, biochemical, or radiologic evidence of cirrhosis or hepatic malignancy. Both immune-active and inactive CHB phases were included, in accordance with established international clinical practice guidelines [9].

#### Liver cirrhosis (LC)

The diagnosis of liver cirrhosis (LC) was primarily based on standardized clinical documentation in electronic medical records, including imaging reports (ultrasound, CT, or MRI), endoscopic findings, clinical signs of portal hypertension, and discharge diagnoses made by attending hepatologists in accordance with established clinical guidelines. Non-invasive fibrosis indices (APRI and FIB-4) were used only as supportive information when imaging or elastography data were unavailable and were not applied as stand-alone diagnostic criteria, consistent with current clinical practice guidelines for the diagnosis of liver cirrhosis [10].

#### Hepatocellular carcinoma (HCC)

HCC was diagnosed according to guidelines [11] based on one of the following:

- Characteristic contrast-enhanced CT/MRI findings (arterial phase hyperenhancement with portal venous or delayed washout);
- Alpha-fetoprotein (AFP) ≥ 400 ng/mL with radiologic suspicion;
- Histopathologic confirmation.

Only HBV-related HCC cases were included.

### Laboratory measurements

#### Autoantibody testing

- ANA was measured using a YHLO chemiluminescence immunoassay. ANA titers ≥ 1:80 (including ≥ 1:160, ≥ 1:320, and higher) were defined as positive.
- Extended autoimmune liver–specific autoantibodies (AMA-M2, SP100, GP210, LKM-1, LC1, SLA) were detected using the Tenfly Phoenix A immunoblot system.
- Anti–dsDNA antibodies were quantified using the Bio-Rad BioPlex 2200 chemiluminescence platform.

#### Liver biochemistry and hematology

Liver enzymes, bilirubin fractions, albumin, and GGT were measured using the Hitachi Labospect 008AS chemistry analyzer. Complete blood counts were obtained using the Sysmex XN-9100 hematology analyzer.

#### HBV-DNA quantification

HBV-DNA levels were measured using a real-time polymerase chain reaction assay (ABI 7500 platform). HBV-DNA values were log10-transformed for statistical analyses due to their non-normal distribution. Values reported as < 20 IU/mL were assigned a value of 20 IU/mL for statistical analysis.

#### Fibrosis indices

APRI and FIB-4 were calculated using standard formulas:

- APRI = (AST / ULN) × 100 / Platelet count (10⁹/L).
- FIB-4 = (Age × AST) / (Platelet count × √ALT).

The upper limit of normal (ULN) for AST was defined according to institutional laboratory standards. APRI and FIB-4 were calculated using previously published formulas [7, 8].

### Statistical analysis

Continuous variables were analyzed using the Mann–Whitney U test or the Kruskal–Wallis test, as appropriate. Categorical variables were compared using the chi-square (χ²) test or Fisher’s exact test. Receiver operating characteristic (ROC) curves were constructed to assess the diagnostic performance of APRI and FIB-4 for identifying advanced liver disease, with area under the curve (AUC) values and 95% confidence intervals calculated. All statistical tests were two-sided, and significance was set at *p* < 0.05. Given the exploratory nature of this study, no formal adjustment for multiple testing was applied. Analyses were performed using SPSS version 26.0 (IBM Corp., Armonk, NY, USA).

## Results

### Patient characteristics

A total of 151 patients with HBV-related liver disease were included in this retrospective analysis, comprising 50 patients with chronic hepatitis B (CHB), 50 with liver cirrhosis (LC), and 51 with hepatocellular carcinoma (HCC). Baseline demographic and laboratory characteristics are summarized in Table 1. With progression of HBV-related disease severity, markers of liver injury and fibrosis showed a stepwise increase across disease stages. In parallel, ANA positivity increased across disease stages, whereas extended autoantibody positivity remained infrequent in all groups. Antiviral therapy status was not compared across disease groups due to incomplete and heterogeneous treatment data.

**Table 1:** Baseline demographic and clinical characteristics of patients with HBV-related liver disease

Variable	CHB (n = 50)	LC (n = 50)	HCC (n = 51)
Age, years (median)	49.5	57.0	60.0
Sex, n (%)	M: 25 (50%) / F: 25 (50%)	M: 28 (56%) / F: 22 (44%)	M: 44 (86.3%) / F: 7 (13.7%)
ALT (U/L), median	50.30	27.05	34.20
AST (U/L), median	27.0	27.5	39.0
Albumin (g/L), median	41.25	39.50	38.20
Total bilirubin (µmol/L), median	13.00	16.05	15.90
Platelets (×10⁹/L), median	199.0	124.0	141.0
HBV-DNA (IU/mL), median^a^	20.0	40.8	33.3

^a^For HBV-DNA values reported as “<20 IU/mL,” a value of 20 IU/mL was used for statistical calculations, consistent with the methods section

### ANA and related antibody positivity across disease stages

**Fig. 1:**
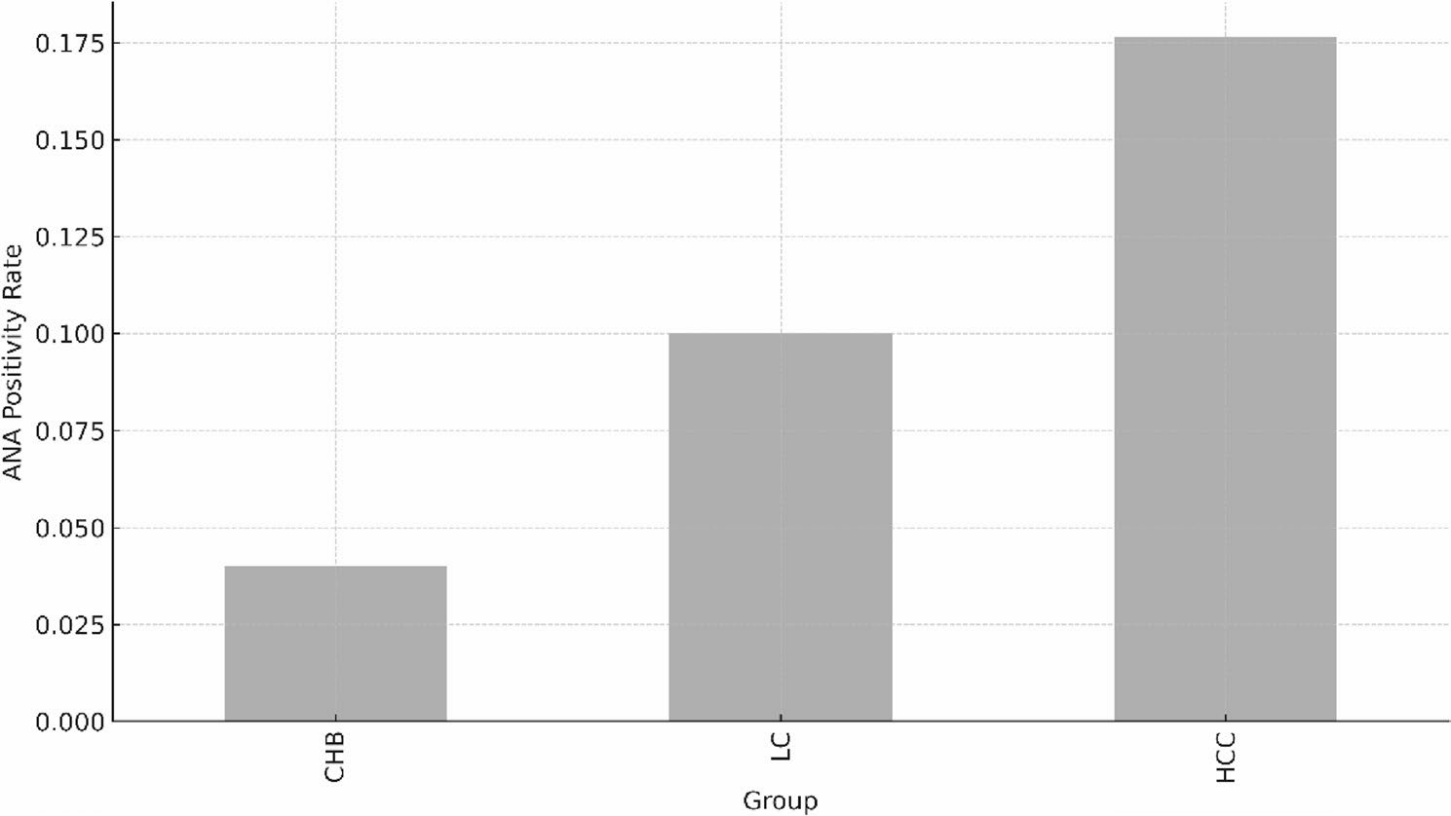
Prevalence of ANA positivity across HBV-related disease stages. The proportion of patients with ANA ≥1:80 increased progressively from chronic hepatitis B (CHB) to liver cirrhosis (LC) and hepatocellular carcinoma (HCC). Most ANA-positive cases were of low titer. Group differences were assessed using the chi-square test or Fisher’s exact test, as appropriate

ANA positivity increased progressively across the spectrum of HBV-related liver disease. The prevalence of ANA positivity was 4.0% in CHB, 10.0% in LC, and 17.6% in HCC, demonstrating a statistically significant increase with advancing disease stage (*p* < 0.05) (Fig. 1). Most ANA-positive cases were of low titer (1:80–1:160). High ANA titers were uncommon: three patients exhibited a titer of 1:320 (one each in the CHB, LC, and HCC groups), and one patient in the HCC group had a titer of 1:640. Owing to the very small number of high-titer cases, no clear stage-specific pattern could be established. Anti-dsDNA antibodies were detected in 5 patients with CHB, 13 patients with LC, and 13 patients with HCC. Detailed results are provided in Supplementary Table S1.

### Extended autoimmune liver–specific autoantibodies

Extended autoimmune liver–specific autoantibodies were infrequently detected across all disease stages. Specifically, AMA-M2 positivity was observed in one patient with liver cirrhosis, SP100 positivity in one patient with chronic hepatitis B, and GP210 positivity in one patient with chronic hepatitis B, one patient with liver cirrhosis, and two patients with hepatocellular carcinoma. No patients tested positive for LKM-1, LC1, or SLA antibodies. No individual exhibited positivity for more than one extended autoantibody. No significant differences were observed among the CHB, LC, and HCC groups. Detailed results are provided in Supplementary Table S1.

### APRI and FIB-4 across disease stages

**Fig. 2:**
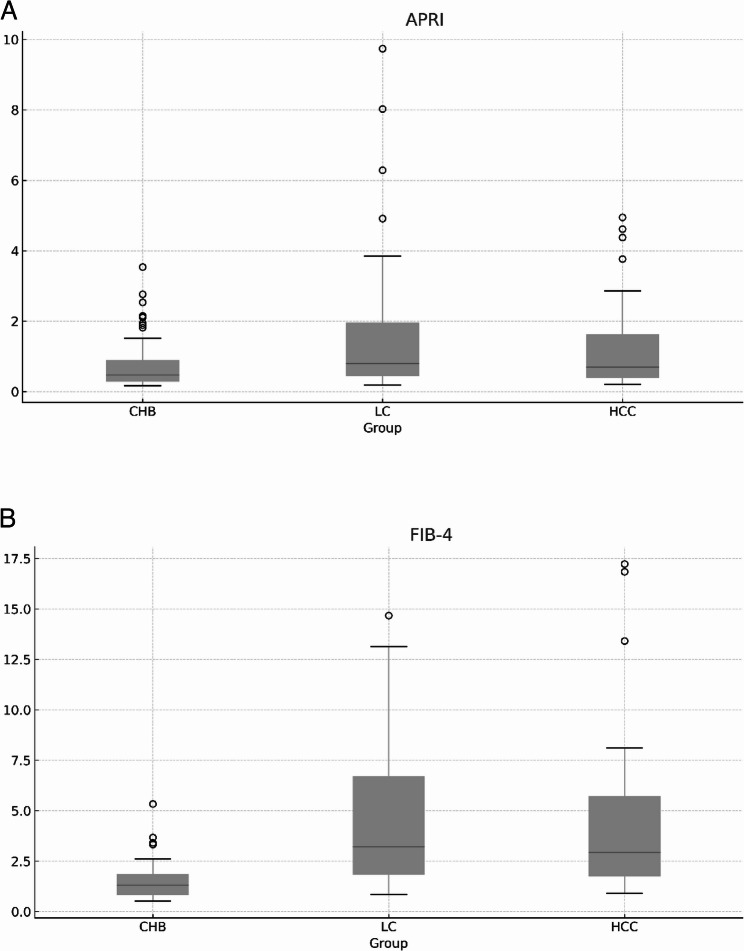
Distribution of APRI values among CHB, LC, and HCC patients. **A**. Distribution of APRI values among CHB, LC, and HCC patients.APRI values showed a stepwise increase from CHB to LC and HCC. Comparisons among groups were performed using the Kruskal–Wallis test. **B**. Distribution of FIB-4 values across HBV-related disease stages.FIB-4 values increased with advancing liver disease and showed clearer separation among CHB, LC, and HCC groups compared with APRI. Comparisons among groups were performed using the Kruskal–Wallis test

APRI and FIB-4 values increased markedly with disease progression from CHB to LC and HCC (Fig. 2A and 2B). The median APRI values were 0.43 in CHB, 1.15 in LC, and 1.80 in HCC, reflecting a continuous elevation across stages. Similarly, median FIB-4 values rose from 1.2 in CHB to 3.0 in LC and 4.8 in HCC. Both noninvasive fibrosis indices differed significantly among the three groups (*p* < 0.001), consistent with worsening liver fibrosis severity.

### Diagnostic performance for advanced liver disease

**Fig. 3:**
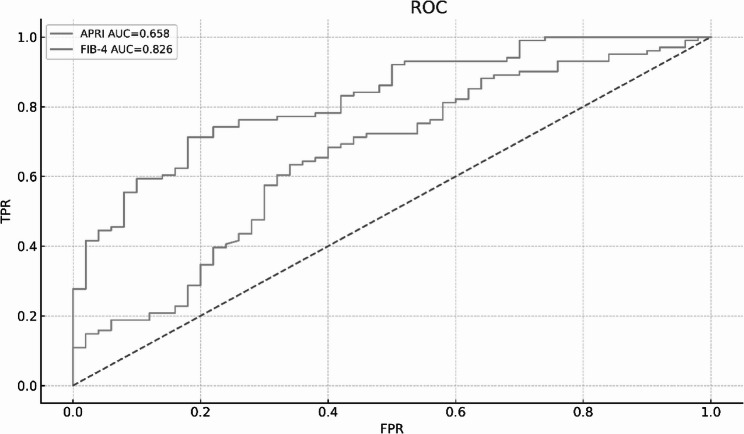
Receiver operating characteristic (ROC) curves of APRI and FIB-4 for identifying advanced liver disease (LC + HCC). ROC analysis demonstrated discriminatory performance of both APRI and FIB-4 for identifying advanced liver disease, with FIB-4 showing a higher area under the curve (AUC)

In this study, advanced liver disease was defined as LC and HCC (LC + HCC). Receiver operating characteristic analysis demonstrated that both APRI and FIB-4 had discriminatory ability for identifying advanced liver disease. The AUC (95% CI) for APRI was 0.66 (0.56–0.75), whereas FIB-4 achieved a higher AUC (95% CI) of 0.83 (0.76–0.89), indicating better diagnostic performance (Fig. 3). Using established cut-off values, APRI (≥ 1.5) demonstrated a sensitivity of 29.7% and a specificity of 80.0% for identifying advanced liver disease, whereas FIB-4 (≥ 3.25) showed a sensitivity of 45.5% and a specificity of 92.0%. Detailed classification performance is provided in Supplementary Table S2.

### ANA positivity and fibrosis indices

ANA positivity did not show a significant correlation with APRI or FIB-4 (*p* > 0.05). ANA-positive patients with elevated FIB-4 values were observed predominantly in the LC and HCC groups, reflecting the distribution of disease stage rather than a direct association between ANA positivity and fibrosis indices. Detailed distributions are shown in Supplementary Table S3.

## Discussion

In this retrospective laboratory-based study, we evaluated autoantibody profiles and noninvasive fibrosis indices across the clinical spectrum of HBV-related liver disease. We observed a progressive increase in ANA positivity from CHB to LC and HCC, accompanied by a rising fibrosis burden as reflected by APRI and FIB-4. In contrast, extended autoimmune liver–specific autoantibodies classically associated with autoimmune liver diseases remained rare across all disease stages, supporting the interpretation that ANA positivity in chronic HBV infection is more likely attributable to nonspecific immune activation rather than true autoimmune liver disease.

These findings are particularly relevant in routine laboratory practice. Isolated low-titer ANA positivity in patients with chronic HBV infection may prompt unnecessary investigations for autoimmune liver disease or empirical immunosuppressive therapy. Our results support a cautious interpretation, emphasizing that ANA positivity in HBV should be evaluated within the broader clinical context rather than used as a stand-alone indicator of autoimmune pathology. In addition, anti-dsDNA antibodies were detected more frequently with disease progression, particularly in advanced liver disease. However, liver-specific autoimmune autoantibodies remained uncommon, reinforcing the concept that autoantibody positivity in HBV-related liver disease primarily reflects nonspecific immune dysregulation. Recent studies have suggested that advanced HCC may exhibit phenotypic features overlapping with autoimmune liver diseases, possibly reflecting profound immune dysregulation in late-stage disease [12]. This phenomenon may partly contribute to the increased frequency of ANA positivity observed in advanced HBV-related liver disease, although our findings remain descriptive.

Extended autoimmune liver–specific autoantibodies, including AMA-M2, GP210, SP100, LKM-1, LC1, and SLA were infrequently detected across all disease categories. This low prevalence is consistent with the high specificity of these markers for autoimmune liver diseases and with previous reports describing their rarity in chronic viral hepatitis [13–15]. From a clinical laboratory perspective, isolated positivity of these autoantibodies in patients with HBV infection may lead to unnecessary diagnostic evaluations for autoimmune overlap syndromes.

Both APRI and FIB-4 demonstrated clear stage-dependent increases and showed discriminatory value for identifying advanced liver disease. These findings are consistent with prior validations of APRI and FIB-4 as noninvasive fibrosis markers in HBV-related liver disease [16, 17]. Notably, FIB-4 exhibited better diagnostic performance, in line with large HBV cohort studies demonstrating its utility in identifying advanced fibrosis and hepatocellular carcinoma risk [18–20]. Beyond statistical performance, FIB-4 is inexpensive, based on universally available laboratory parameters, and can be repeatedly measured over time. In settings where liver biopsy or elastography is not routinely available, FIB-4 may serve as a pragmatic tool for identifying patients who warrant closer surveillance or referral to specialist care.

Importantly, ANA positivity did not show a direct association with APRI or FIB-4, suggesting that immune activation and fibrotic progression may represent largely independent processes in chronic HBV infection [21, 22]. Nevertheless, the clustering of ANA-positive individuals with elevated FIB-4 values within the LC and HCC subgroups suggests that combined interpretation of immunologic and fibrosis markers may provide complementary information when evaluating patients with advanced disease or diagnostically ambiguous presentations.

The strengths of this study include a well-characterized real-world HBV cohort spanning the full spectrum of disease stages (CHB, LC, and HCC), the use of standardized laboratory assays, and the parallel assessment of autoantibody profiles and noninvasive fibrosis indices.

This study has several limitations. First, its retrospective design precludes causal inference. Second, histological confirmation of fibrosis was not available, and noninvasive indices such as FIB-4 cannot fully replace liver biopsy in all clinical scenarios. Third, the absence of longitudinal follow-up data limited assessment of prognostic outcomes, including hepatic decompensation and mortality. Accordingly, the present findings should be interpreted as reflecting laboratory associations rather than causal relationships. In addition, we did not explore quantitative associations between anti-dsDNA antibodies and fibrosis indices, as anti-dsDNA was interpreted primarily as a marker of systemic immune dysregulation rather than a mechanistic indicator of fibrosis progression. Differences in age and sex distributions among the CHB, LC, and HCC groups may have contributed to the observed increase in ANA positivity, and residual confounding cannot be excluded. Finally, because APRI and FIB-4 were used as supportive information for cirrhosis classification in a subset of patients when imaging or elastography data were unavailable, a degree of incorporation bias may be present.

## Conclusion

ANA positivity increases across HBV-related disease stages but appears to primarily reflect nonspecific immune activation rather than true autoimmune liver disease. Extended autoimmune liver–specific autoantibodies are uncommon and provide limited additional diagnostic value in chronic HBV infection. Among non-invasive fibrosis markers, FIB-4 demonstrates better diagnostic performance than APRI for identifying advanced liver disease. Taken together, consideration of autoantibody patterns alongside fibrosis indices may aid laboratory interpretation and support more informed clinical assessment in patients with chronic HBV infection.

## Supplementary Information

Supplementary Material 1.

## Data Availability

Data are available from the corresponding author upon reasonable request.
