# Supplementary material for: Autoantibody profiles and fibrosis indices across HBV-related disease stages: a retrospective laboratory-based study in a tertiary hospital in China
Source: BMC Gastroenterol. 2026 Mar 11;26:238. doi: 10.1186/s12876-026-04718-4 (PMC13088563; doi:10.1186/s12876-026-04718-4)
Supplement: Supplementary file 1 — Supplementary Material 1. [file 12876_2026_4718_MOESM1_ESM.docx]

**Supplementary Table S1. Distribution of extended autoimmune liver–specific autoantibodies and anti-dsDNA across CHB, LC, and HCC groups**

| **Group** | **AMA-M2 positive** | **SP100 positive** | **GP210 positive** | **LKM-1 positive** | **LC1 positive** | **SLA positive** | **dsDNA positive** |
| --- | --- | --- | --- | --- | --- | --- | --- |
| **CHB** | 0 | 1 | 1 | 0 | 0 | 0 | 5 |
| **LC** | 1 | 0 | 1 | 0 | 0 | 0 | 13 |
| **HCC** | 0 | 0 | 2 | 0 | 0 | 0 | 13 |

**Footnote:**

Values represent the number of patients testing positive for each autoantibody in each disease group. Anti-dsDNA antibodies are shown separately from autoimmune liver–specific autoantibodies.

**Supplementary Table S2. Diagnostic performance of APRI and FIB-4 at established cut-off values for advanced liver disease**

| **Index** | **Cut-off** | **Sensitivity (%)** | **Specificity (%)** | **TP** | **FN** | **FP** | **TN** |
| --- | --- | --- | --- | --- | --- | --- | --- |
| **APRI** | 1.5 | 29.7 | 80.0 | 30 | 71 | 10 | 40 |
| **FIB-4** | 3.25 | 45.5 | 92.0 | 46 | 55 | 4 | 46 |

**Footnote:**

Abbreviations: APRI, aspartate aminotransferase-to-platelet ratio index; FIB-4, fibrosis-4 index; TP, true positive; FN, false negative; FP, false positive; TN, true negative. Advanced liver disease was defined as liver cirrhosis and hepatocellular carcinoma (LC + HCC). Sensitivity and specificity were calculated using established cut-off values (APRI ≥1.5 and FIB-4 ≥3.25).

**Supplementary Table S3. Counts of ANA positivity and high FIB-4 values across disease stages**

| **Group** | **ANA positive (n)** | **High FIB-4 (n)** |
| --- | --- | --- |
| **CHB** | 2 | 4 |
| **LC** | 5 | 22 |
| **HCC** | 9 | 24 |

**Footnote:**

“High FIB-4” was defined according to commonly used thresholds indicative of advanced fibrosis. Values represent the number of patients in each disease group meeting the respective criteria and are presented descriptively without implying a direct association between ANA positivity and fibrosis severity.
